# Systolic time intervals derived from electrocardiographic gated intra-renal artery Doppler waveform associated with left ventricular systolic function

**DOI:** 10.1038/srep29293

**Published:** 2016-08-24

**Authors:** Wen-Hsien Lee, Po-Chao Hsu, Chun-Yuan Chu, Szu-Chia Chen, Hung-Hao Lee, Meng-Kuang Lee, Chee-Siong Lee, Hsueh-Wei Yen, Tsung-Hsien Lin, Wen-Chol Voon, Wen-Ter Lai, Sheng-Hsiung Sheu, Ho-Ming Su

**Affiliations:** 1 Graduate Institute of Clinical Medicine, College of Medicine, Kaohsiung Medical University, Kaohsiung, Taiwan; 2 Division of Cardiology, Department of Internal Medicine, Kaohsiung Medical University Hospital, Kaohsiung Medical University, Kaohsiung, Taiwan; 3 Department of Internal Medicine, Kaohsiung Municipal Hsiao-Kang Hospital, Kaohsiung Medical University, Kaohsiung, Taiwan; 4 Faculty of Medicine, College of Medicine, Kaohsiung Medical University, Kaohsiung, Taiwan

## Abstract

The aims of this study were to investigate the correlation between renal and cardiac STIs, including pre-ejection period (PEP), ejection time (ET), and PEP/ET, and to assess the diagnostic values of renal STIs in predicting left ventricular ejection fraction (LVEF) <50%. The cross sectional observation study enrolled 230 participants. The renal STIs, including renal PEP (rPEP), renal ET (rET), and rPEP/rET, were measured from electrocardiographic gated renal Doppler ultrasound and cardiac PEP, ET, and PEP/ET were measured from echocardiography. Renal STIs were correlated with cardiac STIs (all P < 0.001). Multivariate analyses showed that rPEP/rET was independently associated with LVEF (unstandardized coefficient β = −0.116, P = 0.046) and LVEF <50% (odds ratio = 2.145, per 0.11 increase; P = 0.017). The areas under the curve for rPEP, 1/rET, and rPEP/rET in predicting LVEF <50% were 0.773, 0.764, and 0.821, respectively. The sensitivity and specificity of rPEP/rET > 0.46 in prediction of LVEF <50% were 76.7% and 78.1%, respectively. Our study demonstrated that the novel parameters of renal STIs were significantly associated with cardiac STIs. However, the clinical application of renal STIs needs to be investigated in future studies.

Periods of cardiac cycles, particularity left ventricular systolic time intervals (STIs), were established for fifty years ago^1^,^2^. STIs, including pre-ejection period (PEP), ejection time (ET), and ratio of PEP to ET measured from invasive intra-cardiac hemodynamic technique, non-invasive arterial pulse recording, or echocardiography, were useful parameters of cardiac systolic performance^1^,^2^,^3^. PEP measured from onset of electrocardiographic QRS complex to aortic valve opening is meaning of the time interval from onset of ventricular depolarization to the start of ventricular ejection. ET is the time period from beginning to termination of ventricular ejection or arterial upstroke^3^,^4^. Increased PEP, decreased ET, and increased PEP/ET have been reported to be significantly correlated with decreased left ventricular ejection fraction (LVEF)^5^,^6^,^7^. STIs were widely measured from pulse Doppler or tissue Doppler echocardiography^8^,^9^. Echocardiography derived STIs were investigated in patients with heart failure, coronary diseases, or under cardiac resynchronization therapy^10^,^11^,^12^. STIs were also measured from non-invasive arterial pulse recording, phonocardiography, and electrocardiography. Our previous study demonstrated that brachial PEP, brachial ET, and brachial PEP/ET measured from an automatic device were significant associated with LVEF^13^. Recently, Polak *et al.* found ET measured from only Doppler waveform of carotid artery was also significantly associated with LVEF^14^. However, Polak *et al.* did not investigate PEP and PEP/ET from arterial Doppler waveform. The applications of STIs are makers of global cardiac systolic function and predictors of adverse cardiac outcome in patients with heart failure^3^,^4^,^15^.

Owing to high cardiovascular morbidity and mortality, the increasing number of patients with chronic kidney disease (CKD) is an important healthcare issue in the world^16^,^17^. Renal ultrasonography is an useful image tool in noninvasive evaluation of renal anatomic and vascular information in patients with renal injury^18^. In addition to conventional gray-scale image, renal Doppler ultrasound can help to evaluate intra-renal vascular information. The popular parameter measured from renal Doppler ultrasound is renal resistive index (RI). Renal RI can reflect vascular resistance and serve as a predictor of renal damage and poor cardiovascular outcome^19^,^20^,^21^. Furthermore, renal RI was associated with left ventricular diastolic dysfunction^20^, but not associated with LVEF^22^.

Although STIs measured from Doppler ultrasound were associated with global cardiac systolic function, there was no study to evaluate whether renal STIs measured from renal Doppler ultrasound were also associated with cardiac systolic function. Hence, the aims of this study were to investigate the relationship between renal STIs measured from real-time internal electrocardiographic (ECG) gated renal Doppler ultrasound and cardiac STIs measured from echocardiography and to assess the diagnostic values of renal STIs in prediction of LVEF <50%^23^.

## Methods

**Figure 1:**
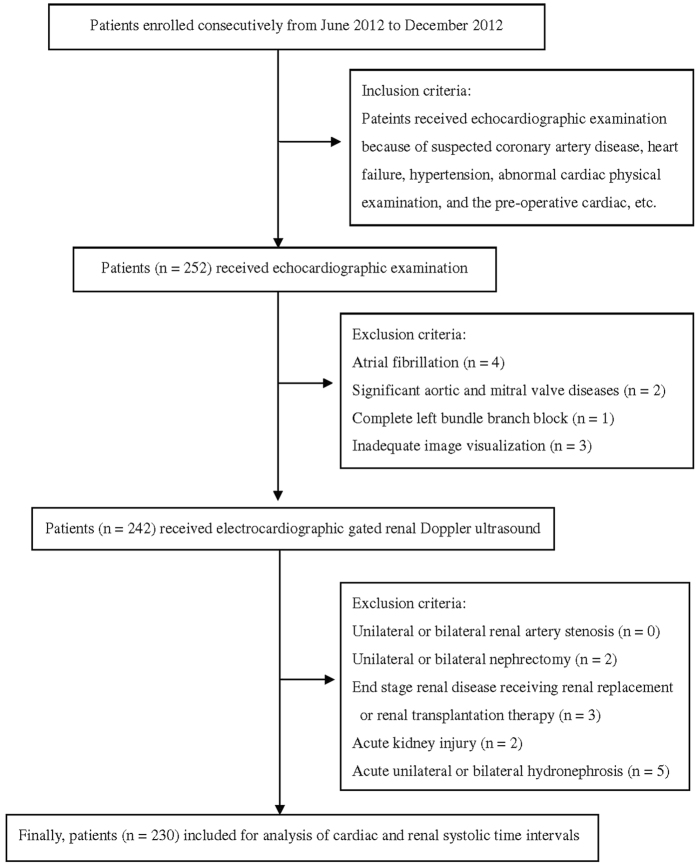
Flow chart of study patients.

This is a cross-sectional observation study enrolled participants who received echocardiographic examination due to suspected cardiovascular diseases in a regional hospital in Taiwan from June 2012 to December 2012 (Fig. 1). Patients with atrial fibrillation (number, n = 4), significant aortic or mitral valve diseases (n = 2), left bundle branch block (n = 1), inadequate image visualization (n = 3), history of unilateral or bilateral renal artery stenosis (n = 0), unilateral or bilateral nephrectomy (n = 2), end stage renal disease receiving renal replacement or renal transplantation therapy (n = 3), acute kidney injury (n = 2), and acute unilateral or bilateral hydronephrosis (n = 5) were excluded.

### Ethics statement

The study methods were carried out in accordance with the approved guidelines. The study protocols were approved by the institutional review board committee of the Kaohsiung Medical University Hospital (KMUHIRB-E(II)-20160015). Written informed consent was obtained from all subjects.

### Renal Doppler ultrasound study

**Figure 2:**
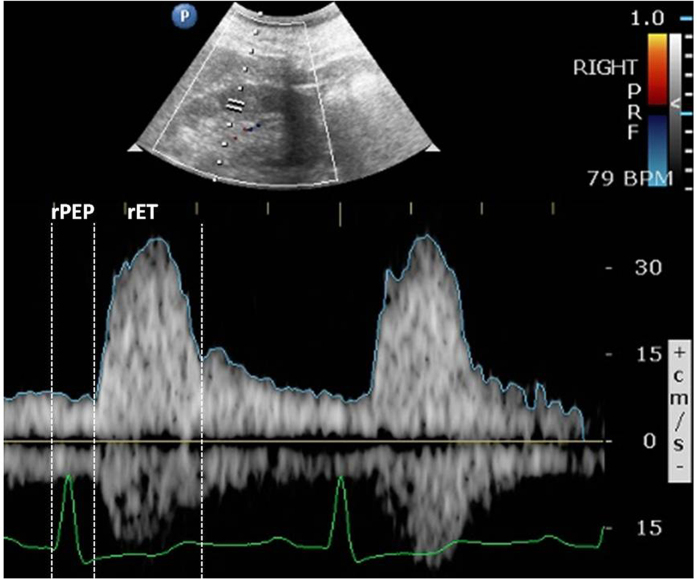
The renal pre-ejection period (rPEP) was measured from the onset of the QRS complex to the foot of the renal pulse Doppler waveform. The renal ejection time (rET) was measured from the foot to the dicrotic notch of the renal pulse Doppler waveform.

Ultrasonographic examinations were performed using multi-functional duplex Doppler ultrasonography with a CX50 (Philips) ultrasound machine with a 2.5-MHz pulsed Doppler frequency and a 3.5-MHz convex array transducer. The image of the kidneys was determined by B-mode and renal blood flow was visualized with color-Doppler sonography superimposed on B-mode image while the patient was in the supine position. We applied internal ECG signal into Doppler ultrasound. Then, intra-renal Doppler signals were obtained from the arcuate arteries at the cortico-medullary junction. The renal RI was calculated as (peak systolic velocity – minimum diastolic velocity)/peak systolic velocity^24^. The renal PEP (rPEP) was measured from the onset of the QRS complex to the foot of the renal pulse Doppler waveform. The renal ET (rET) was measured from the foot to the dicrotic notch of the renal pulse Doppler waveform (Fig. 2). The rPEP and rET were determined three times for each kidney and then the values from bilateral kidneys were averaged to obtain the mean value for later analysis. All measurements were performed by one experienced physician who was blinded to the other data of the subjects.

### Echocardiographic assessment

A single experienced cardiologist performed all echocardiographic examination and acquired image using the Vivid 7 (General Electrics). Left ventricular internal diameter (LVID), interventricular septal wall thickness (IVST), left ventricular posterior wall thickness (LVPWT), trans-mitral E wave velocity (E), E-wave deceleration time, trans-mitral A wave velocity and early diastolic mitral velocity (Ea), were measured by standard chamber quantification and measurement^13^,^25^. We calculated LVEF and Left ventricular mass by the modified Simpson’s method and the Devereux-modified method, respectively^26^. Left ventricular mass index was calculated by dividing left ventricular mass by body surface area. PEP was measured from the onset of the QRS complex on the electrocardiogram to the onset of systolic flow from the left ventricular outflow tract (LVOT). ET was measured from the onset to the end of LVOT systolic flow^13^. The PEP and ET were obtained from 3 consecutive beats and then the data were averaged to give the mean value for later analysis. All echocardiographic parameters were acquired from 3 continued beats and measured from offline EchoPAC software by a single experienced cardiologist.

### Collection of demographic, medical, and laboratory data

Baseline medication, personal characteristic, and laboratory data were collected from medical records. The value of estimated glomerular filtration rate (eGFR) was calculated by the equation of Modification of Diet in Renal Disease study^27^. Participants with CKD were defined as those with evidence of kidney damage lasting for more than 3 months^28^ and an eGFR <60 ml/min/1.73m^2^.

### Reproducibility

Thirty patients were randomly selected for evaluation of the interobserver variability of renal STIs measurement by two independent observers. To obtain intraobserver variability, the same measurement was repeated 1 week apart. Mean percentage error was calculated as the absolute difference divided by the average of the two observations.

### Statistical analyses

Baseline data are expressed as percentages or mean ± standard error. The differences in items between renal and echocardiographic STIs were checked by Student’s t-test. The relationship between two continuous variables was assessed using a bivariate correlation method (Pearson’s correlation). Bland-Altman plots were used to assess the agreements between renal and echocardiographic STIs. The regression of the average and the difference between renal and echocardiographic STIs (renal STIs minus echocardiographic STIs) was analyzed. Significant variables in the univariate analysis were further analyzed by multivariate stepwise linear regression and backward logistic regression to identify the determinants of LVEF and LVEF <50%, respectively. Receiver operating characteristic (ROC) curve was constructed for the prediction of LVEF <50%. A difference was considered significant if the P value was less than 0.05. Statistical analysis was performed using SPSS version 20.0 (SPSS Inc., Chicago, IL, USA).

## Results

A total of 230 participants (age 64.0 ± 12.2 years, male 61.5%) enrolled in this study. Table 1 shows the clinical, echocardiographic, and renal Doppler ultrasonographic characteristics of these patients. The mean values of renal RI, rPEP, rET, and rPEP/rET were 0.69 ± 0.08, 123.7 ± 23.7 ms, 303.0 ± 36.8 ms, and 0.42 ± 0.11, respectively. The mean values of PEP, ET, and PEP/ET were 67.7 ± 15.1 ms, 294.8 ± 34.5 ms, and 0.23 ± 0.07, respectively.

Table 2 displays the determinants of LVEF and LVEF <50% according to univariate analysis in the study subjects. Male gender, diabetes, congestive heart failure, increased heart rate, increased triglyceride, decreased eGFR, increased glucose, use of β-blockers, no use of calcium channel blockers, use of diuretics, increased rPEP, decreased rET, increased rPEP/rET, increased PEP, decreased ET, and increased PEP/ET were significantly associated with decreased LVEF in the univariate linear analysis (P ≦ 0.017). Additionally, male gender, diabetes, congestive heart failure, increased heart rate, increased triglyceride, decreased eGFR, increased glucose, use of β-blockers, use of diuretics, increased rPEP, decreased rET, increased rPEP/rET, increased PEP, decreased ET, and increased PEP/ET were significantly associated with LVEF <50% in the univariate logistic analysis (P ≦ 0.032).

Table 3 shows the determinants of LVEF and LVEV <50% by multivariable linear and logistic analyses. In the linear and logistic multivariate models, covariates included the significant variables in univariate analysis (in Table 2) plus rPEP, rET, rPEP/rET and PEP, ET, PEP/ET, respectively. In multivariable linear model, rPEP/rET (unstandardized coefficient β = −0.116, P = 0.046), PEP (unstandardized coefficient β = −0.002, P < 0.001), and PEP/ET (unstandardized coefficient β = −0.508, P < 0.001) were significantly associated with increased LVEF. In multivariable logistic model, rET (odds ratio = 0.970, P = 0.008), rPEP/rET (odds ratio = 2.145 per 0.11 increase, P = 0.017), PEP (odds ratio = 1.105, P = 0.007), ET (odds ratio = 0.970, P = 0.028), and PEP/ET (odds ratio = 5.114 per 0.07 increase, P = 0.004) were significantly associated with LVEF <50%.

**Figure 3:**
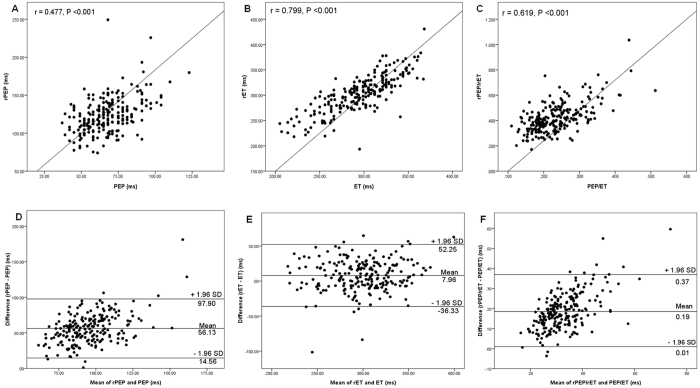
The scatter plots between pre-ejection period (PEP) and renal PEP (rPEP) (**A**), ejection time (ET) and renal ET (rET) (**B**), and PEP/ET and rPEP/rET (**C**) and Bland-Altman plots of PEP and rPEP (**D**), ET and rET (**E**), and PEP/ET and rPEP/rET (**F**) in all subjects.

In the Pearson’s correlation analyses, LVEF was significantly correlated with rPEP (r = −0.338), rET (r = 0.317), rPEP/rET (r = −0.430), PEP (r = −0.477), ET (r = 0.251), and PEP/ET (r = −0.517) (all P < 0.001). However, LVEF was not significantly associated with renal RI (r = −0.046, P = 0.482). Figure 3 shows the scatter plots between rPEP and PEP (r = 0.477, P < 0.001) (Fig. 3A), rET and ET (r = 0.799, P < 0.001) (Fig. 3B), and rPEP/rET and PEP/ET (r = 0.619, P < 0.001) (Fig. 3C). To assess the agreement between renal and echocardiographic STIs, Bland-Altman plots were produced. The mean value of rPEP minus PEP was 56.13 ms and the 95% limit of agreement was 14.56 to 97.90 ms (Fig. 3D). The mean value of rET minus ET was 7.96 ms and the 95% limit of agreement was −36.33 to 52.25 ms (Fig. 3E). Finally, the mean value of rPEP/rET minus PEP/ET was 0.19 and the 95% limit of agreement was 0.01 to 0.37 (Fig. 3F).

**Figure 4:**
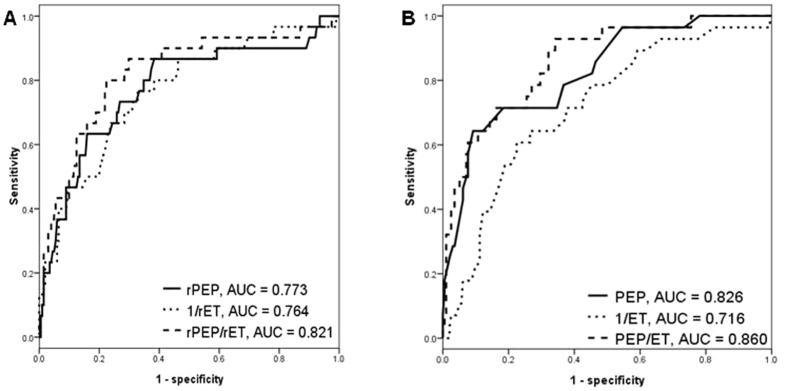
The areas under the curve (AUCs) for renal pre-ejection period (rPEP), 1/renal ejection time (rET), and rPEP/rET measured from renal Doppler ultrasound (**A**) and pre-ejection period (PEP), 1/ejection time (ET), and PEP/ET measured from echocardiography (**B**) in prediction of left ventricular ejection fraction <50%.

Figure 4 shows the ROC curves for rPEP, 1/rET, and rPEP/rET (Fig. 4A) and PEP, 1/ET, and PEP/ET (Fig. 4B) in prediction of LVEF <50%. The areas under the curve (AUCs) for rPEP, 1/rET, and rPEP/rET in prediction of LVEF <50% were 0.773, 0.764, and 0.821, respectively (all P < 0.001). In addition, the AUCs for PEP, ET, and PEP/ET in prediction of LVEF <50% were 0.826, 0.716, and 0.860, respectively (all P < 0.001).

Table 4 shows the statistical values of rPEP, rET, rPEP/rET, PEP, ET, and PEP/ET in prediction of LVEF <50%.

The intra-observer mean percentage errors (95% confidence interval) for renal PEP, renal ET, and renal PEP/ET measurement were 3.7 ± 4.1% (<0.1%, 13.9%), 2.0 ± 1.9% (<0.1%, 6.3%), and 5.0 ± 5.3% (<0.1%, 14.3%), respectively. The inter-observer mean percentage errors (95% confidence interval) for renal PEP, renal ET, and renal PEP/ET measurement were 4.1 ± 5.3% (<0.1%, 14.6%), 3.3 ± 1.8% (<0.1%, 8.9%), and 8.4 ± 5.7% (<0.1%, 19.8%), respectively.

## Discussion

In the present study, we found that renal STIs derived from ECG gated renal Doppler ultrasonography were significantly associated with cardiac STIs measured from echocardiography and were useful in identification of patients with LVEF <50%.

Cardiac STIs affected by many physiological and pharmacologic factors are well-established parameters in assessment of global cardiac performance^3^,^29^,^30^,^31^,^32^. In general, cardiac STIs may be affected by several medications, such as digitalis glycosides, catecholamines, propranolol, amyl nitrate, and calcium gluconate, through positive or negative inotropic effects^31^,^32^. The physiological meaning of cardiac PEP is composed of isovolumetric contraction time and electromechanical delay, i.e. time interval between initiation of ventricular depolarization and aortic valve opening^3^,^29^,^33^. Regardless of causes of heart failure, increased cardiac PEP is resulted from a decreased rate of left ventricular pressure rise (dP/dt) during isovolumic contraction period^3^,^34^. The PEP may prolong when patients with impairment of cardiac contractility, left bundle branch block, use of negative inotropic agents, decreased preload status, or increased afterload pressure^31^,^32^. The PEP may shorten when patients with aortic valve disease, use of positive inotropic agents, increased preload status, or decreased afterload pressure. Although the physiological meaning of cardiac ET is the period from beginning to finishing of left ventricular ejection, the factors contributed to ET are more complex^3^. The length of PEP, preload condition, strength of myocardial fiber, and inotropic agents are associated with ET period^3^,^29^. Unlike PEP, which respectively became shorten and lengthen by positive and negative inotropic agents, ET became shorten both by positive and negative inotropic agents^35^,^36^. Although Polak *et al.* showed ET measured from Doppler waveform of carotid artery was significantly associated with LVEF, they did not further evaluate their relationship by multivariate analysis^14^. In this study, all the parameters of renal STIs were correlated with LVEF and useful in prediction of LVEF <50% in the univaiable analyses. Because gender, heart rate, clinical comorbidity, and medication use might influence STIs, we also assessed the determinants of renal STIs from different multivariate models^3^,^13^,^31^. Instead of rPEP and rET, rPEP/rET was still an independent determinant of LVEF and useful in prediction of patients with LVEF <50% in the multivariable analyses.

Although renal Doppler ultrasound was a popular image modality for evaluation of intra-renal hemodynamics, there were few studies researching the relationship between parameters of renal Doppler ultrasound and cardiac systolic function^20^,^37^. In our present study, renal RI was not significantly associated with LVEF. Hence, renal RI might be not a useful parameter in identification of patients with impaired left ventricular systolic function. Additionally, in the present study, cardiac PEP was calculated from onset of QRS to beginning of flow of left ventricular outflow tract. However, rPEP was calculated from onset of QRS to the foot of renal Doppler waveform. Because of the delay of arterial pulse wave from aorta to renal arcuate artery, rPEP was longer (mean difference 56.13 ms) than cardiac PEP in the present study. The physiological meaning of rPEP is composed of isovolumetric contraction time, electromechanical delay, and transmission of arterial pulse wave, i.e. time interval from initiation of ventricular depolarization to the foot of renal pulse Doppler waveform. Hence, renal STIs may be influenced by cardiac systolic function and arterial stiffness.

There were several different measurement methods of STIs. In the method developed by *Weissle et al.*, they simultaneously recorded carotid pulse tracing, phonocardiography, and electrocardiography and then calculated STIs^1^. Recently, a radial artery tonometry was also used to evaluate STIs. In this method, they used high-fidelity pressure transducer, electrocardiography, and special software program to measure STIs^38^. In addition, brachial STIs could be automatically measured from an ABI-form device by an oscillometric method^13^. In the above methods, the high quality and complex equipments were needed to acquire the STIs. In the present study, when performing the renal echo, we used internal ECG signal and renal Doppler waveform to measure renal STIs. This method did not require additional complex equipment and software. Hence, our measurement of renal STIs had several advantages including cost-effectiveness, no need of extra operation, and renal-time calculation. Although echocardiography has been widely used for diagnosis and assessment of global cardiac function, the measurement of LVEF is difficult in patients with poor image visualization, extreme obesity, and severe pulmonary diseases^33^. Cardiac STIs and perhaps renal STIs were alternative parameters for evaluation of global left ventricular systolic function in these patients^33^. Hence, using renal Doppler ultrasonography, in addition to calculation RI to evaluate intra-renal vascular information, we can extra measure renal STIs to roughly assess global left ventricular systolic function.

## Study limitations

There were several limitations to our study. First, our study was a cross-sectional observation design and only enrolled small number of cases in one regional hospital, which might limit study generality and cause selected bias. Second, due to lack of laboratory biomarkers of heart failure^39^, such as brain natriuretic peptide, N-terminal pro-brain natriuretic peptide, galectin-3, or N-terminal propeptide of procollagen type III, we did not know the relationship between those biomarkers and renal STIs. Finally, lack of echocardiographic parameters of right ventricle^40^, we did not recognize the associations between renal STIs and right ventricular function.

## Conclusions

Our study demonstrated that the novel parameters of renal STIs were significantly associated with cardiac STIs. Additionally, rPEP/rET was associated with LVEF and useful in prediction of patients with LVEF <50%. However, the clinical application of renal STIs needs to be investigated in future large-scale studies.

## Additional Information

**How to cite this article**: Lee, W.-H. *et al.* Systolic time intervals derived from electrocardiographic gated intra-renal artery Doppler waveform associated with left ventricular systolic function. *Sci. Rep.* **6**, 29293; doi: 10.1038/srep29293 (2016).

## Figures and Tables

**Table 1:** Clinical, renal Doppler ultrasonographic, and echocardiographic characteristics of study patients.

	All patients (number = 230)
Clinical characteristics	
Age (year)	64.0 ± 12.2
Male gender (%)	61.5
Diabetes mellitus (%)	32.5
Hypertension (%)	73.2
Coronary artery disease (%)	16.9
Stroke (%)	8.7
Congestive heart failure (%)	16.5
Systolic blood pressure (mmHg)	133.7 ± 18.6
Diastolic blood pressure (mmHg)	75.8 ± 11.4
Heart rate (min^−1^)	68.8 ± 11.7
Body mass index (kg/m^2^)	26.4 ± 3.8
Triglyceride (mg/dL)	135.4 ± 77.6
Total cholesterol (mg/dL)	192.5 ± 20.7
eGFR (mL/min/1.73 m^2^)	60.7 ± 20.7
Chronic kidney disease (%)	37.1
Hemoglobin (g/dL)	13.4 ± 2.0
ACEI use (%)	18.2
ARB use (%)	43.7
β-blocker use (%)	44.2
Calcium channel blocker use (%)	45.9
Diuretics use (%)	32.5
Renal Doppler ultrasound	
Renal resistive index	0.69 ± 0.08
rPEP (ms)	123.7 ± 23.7
rET (ms)	303.0 ± 36.8
rPEP/rET	0.42 ± 0.11
Echocardiographic data	
PEP (ms)	67.7 ± 15.1
ET (ms)	294.8 ± 34.5
PEP/ET	0.23 ± 0.07
LAD (mm)	36.8 ± 6.1
LVEDD (mm)	51.4 ± 7.3
LVESD (mm)	33.5 ± 9.3
LVMI (g/m^2^)	143.5 ± 45.5
LVEF (%)	64.0 ± 12.7
E (cm/s)	68.7 ± 19.7
EDT (ms)	216.3 ± 68.3
Ea (cm/s)	7.8 ± 2.4
E/Ea	9.8 ± 4.6

Abbreviations. A, transmitral A wave velocity; ACEI: angiotensin converting enzyme inhibitor; ARB: angiotensin II receptor blocker; E, transmitral E wave velocity; Ea, early diastolic mitral velocity; eGFR: estimated glomerular filtration rate; ET, ejection time; LAD, left atrial diameter; LVMI, left ventricular mass index; LVEF, left ventricular ejection fraction; LVEDD: left ventricular end-diastolic dimension; LVESD: left ventricular end-systolic dimension; ms, millisecond; PEP, pre-ejection period; rET, renal ejection time; rPEP, renal pre-ejection period.

**Table 2:** Determinants of LVEF and LVEF <50% by univariate analysis in all patients.

Variables	LVEF		LVEF <50%	
	Unstandardizedcoefficient β (95% CI)	P value	Odds ratio (95% CI)	P value
Clinical characteristics				
Age (year)	0.001 (−0.001, 0.003)	0.059	0.982 (0.952, 1.013)	0.264
Male gender	−0.060 (−0.093, −0.027)	<0.001	2.790 (1.093, 7.124)	0.032
Diabetes mellitus	−0.087 (−0.121, −0.054)	<0.001	6.395 (2.759, 14.824)	<0.001
Hypertension	−0.011 (−0.048, 0.026)	0.553	1.534 (0.596, 3.952)	0.375
Stroke	0.020 (−0.031, 0.071)	0.445	0.233 (0.030, 1.787)	0.161
Congestive heart failure	−0.226 (−0.259, −0.193)	<0.001	49.867 (17.665, 140.768)	<0.001
Systolic blood pressure (per 1 mmHg)	−0.001 (−0.001, 0.001)	0.885	0.998 (0.978, 1.019)	0.862
Diastolic blood pressure (per 1 mmHg)	−0.002 (−0.003, −0.001)	0.009	1.026 (0.994, 1.059)	0.110
Heart rate (per 1 min−^1^)	−0.003 (−0.004, −0.002)	<0.001	1.059 (1.025, 1.094)	0.001
Body mass index (per 1 kg/m^2^)	0.002 (−0.003, 0.006)	0.477	0.904 (0.811, 1.008)	0.069
Triglyceride (per 1 mg/dL)	−0.001 (−0.002, −0.001)	<0.001	1.008 (1.003, 1.013)	0.002
Total cholesterol (per 1 mg/dL)	<0.001 (−0.001, <0.001)	0.963	1.002 (0.992, 1.011)	0.732
eGFR (per 1 mL/min/1.73 m^2^)	0.001 (<0.001, 0.002)	0.002	0.963 (0.944, 0.982)	<0.001
Glucose (per 1 mg/dl)	−0.001 (−0.002, −0.001)	0.011	1.010 (1.002, 1.018)	0.014
Hemoglobin (per 1 g/dL)	0.005 (−0.004, 0.014)	0.285	0.113 (0.683, 1.041)	0.113
ACEI use	−0.005 (−0.048, 0.038)	0.812	1.153 (0.439, 3.024)	0.773
ARB use	0.016 (−0.017, 0.049)	0.350	0.706 (0.320, 1.560)	0.390
β-blocker use	−0.054 (−0.087, −0.022)	0.001	3.486 (1.520, 7.994)	0.003
Calcium channel blocker use	0.040 (0.007, 0.072)	0.017	0.464 (0.203, 1.062)	0.069
Diuretics use	−0.079 (−0.113, −0.046)	<0.001	6.236 (2.692, 14.447)	<0.001
Renal Doppler ultrasound				
Renal resistive index	−0.070 (−0.266, 0.126)	0.482	0.059 (0.832, 13914.355)	0.059
rPEP (per 1 ms)	−0.002 (−0.002, −0.001)	<0.001	1.040 (1.021, 1.060)	<0.001
rET (per 1 ms)	0.001 (0.001, 0.002)	<0.001	0.970 (0.958, 0.983)	<0.001
rPEP/rET	−0.474 (−0.604, −0.345)	<0.001	3.203 (2.075, 4.942)	<0.001
Echocardiography				
PEP (per 1 ms)	−0.004 (−0.005, −0.003)	<0.001	1.100 (1.063, 1.138)	<0.001
ET (per 1 ms)	0.001 (<0.001, 0.001)	<0.001	0.979 (0.968, 0.991)	0.001
PEP/ET	−1.002 (−1.220, −0.783)	<0.001	4.866 (2.794, 8.472)	<0.001

Abbreviations. ACEI: angiotensin converting enzyme inhibitor; ARB: angiotensin II receptor blocker; CI, confidence interval; eGFR: estimated glomerular filtration rate; ET, ejection time; LVEF, left ventricular ejection fraction; PEP, pre-ejection period; rET, renal ejection time; rPEP, renal pre-ejection period. In logistic regression analysis, an increase of 1 unit of rPEP/rET and PEP/ET was equal to an increase of 1 standard deviation (0.11 and 0.07, respectively).

**Table 3:** Determinants of LVEF and LVEF <50% in multivariate analysis in all study patients.

Variables	LVEF		LVEF <50%	
	Unstandardized coefficient β (95% CI)	P value	Odds ratio (95% CI)	P value
rPEP (per 1 ms)	–		1.023 (0.999, 1.048)	0.056
rET (per 1 ms)	–		0.970 (0.949, 0.992)	0.008
rPEP/rET	−0.116 (−0.230, −0.002)	0.046	2.145 (1.145, 4.021)	0.017
PEP (per 1 ms)	−0.002 (−0.003, −0.001)	<0.001	1.105 (1.028, 1.189)	0.007
ET (per 1 ms)	–		0.970 (0.945, 0.997)	0.028
PEP/ET	−0.508 (−0.721, −0.296)	<0.001	5.114 (1.695, 15.424)	0.004

Abbreviations. CI, confidence interval; ET, ejection time; LVEF, left ventricular ejection fraction; PEP, pre-ejection period; rET, renal ejection time; rPEP, renal pre-ejection period. In logistic regression analysis, an increase of 1 unit of rPEP/rET and PEP/ET was equal to an increase of 1 standard deviation (0.11 and 0.07, respectively). In linear and logistic multivariate models, covariates included the significant variables in univariate analysis (in Table 2) plus rPEP, rET, rPEP/rET and PEP, ET, PEP/ET, respectively.

**Table 4:** The statistical values of rPEP, rET, rPEP/rET, PEP, ET, and PEP/ET in prediction of LVEF <50%.

	Odds ratio (95% CI)	P value	Sensitivity	Specificity	Accuracy
rPEP > 139.4 ms	7.965 (3.489, 18.181)	<0.001	63.3	83.6	73.5
rET < 294.1 ms	6.067 (2.562, 14.370)	<0.001	70.0	83.6	76.8
rPEP/rET > 0.46	13.956 (5.376, 36.230)	<0.001	76.7	78.1	77.4
PEP > 76.50 ms	10.811 (4.420, 26.444)	<0.001	71.4	81.6	76.5
ET <281.7 ms	5.019 (2.177, 11.573)	<0.001	64.3	73.5	68.9
PEP/ET > 0.27	12.891 (5.225, 31.804)	<0.001	71.4	83.2	77.3

Abbreviations. CI, confidence interval; LVEF, left ventricular ejection fraction; PEP, pre-ejection period; ET, ejection time; rET, renal ejection time; rPEP, renal.
